# Association of Toll-Like Receptor Signaling and Reactive Oxygen Species: A Potential Therapeutic Target for Posttrauma Acute Lung Injury

**DOI:** 10.1155/2010/916425

**Published:** 2010-07-13

**Authors:** Meng Xiang, Janet Fan, Jie Fan

**Affiliations:** ^1^Department of Surgery, School of Medicine, University of Pittsburgh, Pittsburgh, PA 15260, USA; ^2^Department of Pathophysiology, Fudan University, Shanghai 200032, China; ^3^Department of Medicine, School of Medicine, University of Pittsburgh, Pittsburgh, PA 15260, USA; ^4^Surgical Research, VA Pittsburgh Healthcare System, University Drive, Pittsburgh, PA 15240, USA

## Abstract

Acute lung injury (ALI) frequently occurs in traumatic patients and serves as an important component of systemic inflammatory response syndrome (SIRS). Hemorrhagic shock (HS) that results from major trauma promotes the development of SIRS and ALI by priming the innate immune system for an exaggerated inflammatory response. Recent studies have reported that the mechanism underlying the priming of pulmonary inflammation involves the complicated cross-talk between Toll-like receptors (TLRs) and interactions between neutrophils (PMNs) and alveolar macrophages (AM*ϕ*) as well as endothelial cells (ECs), in which reactive oxygen species (ROS) are the key mediator. This paper summarizes some novel mechanisms underlying HS-primed lung inflammation focusing on the role of TLRs and ROS, and therefore suggests a new therapeutic target for posttrauma ALI.

## 1. Introduction

Trauma is the fifth leading cause of death among all age groups in the United States and is the leading cause of death among people less than 45 years of age [1]. Hemorrhagic shock (HS) that results from major trauma promotes the development of systemic inflammatory response syndrome (SIRS) by priming the innate immune system for an exaggerated inflammatory response [2]. The lung is an important target organ for systemic inflammatory mediators released after severe infection [3, 4] and trauma [5–7], and thus acute lung injury (ALI) frequently occurs in traumatic patients and serves as an important component of SIRS [8]. Over the past decade, despite improvements in supportive care, ALI is still associated with a high mortality rate ranging from 26 to 35 percent [9]. Resuscitated HS promotes the development of ALI by priming an amplified inflammatory response to a second stimulus, the so-called “two-hit hypothesis” [10]. However, the mechanism underlying HS-primed inflammation has yet to be fully determined.

Morphologically, ALI is manifested by alveolar and interstitial fluid accumulation, alveolar hemorrhage, fibrin deposition, and lung neutrophil sequestration. The accumulation of neutrophils (PMNs) in the lung vasculature, interstitium, and alveolar space is considered a critical event and has been the target of various preventative strategies. In our laboratory, a simplified animal model of the “two-hit” paradigm, as an example of human diseases, has been used to address the mechanisms of HS-primed PMNs migration and lung inflammation [4]. In this model, animals are subjected to a nonsevere resuscitated HS (hypotension at 40 mmHg for 1 h), followed by a small intratracheal dose of lipopolysaccharides (LPSs). While neither shock nor LPS alone induced injury, the combination caused lung PMNs accumulation and increased ^125^I-albumin transpulmonary flux [11], suggesting that the mechanisms underlying the HS-primed ALI involve cross talk between Toll-like receptors (TLRs) and interactions between PMNs and alveolar macrophages (AM*ϕ*) as well as endothelial cells (ECs), in which reactive oxygen species (ROSs) are a key mediator. The purpose of this paper is to summarize some novel mechanisms underlying HS-primed lung inflammation focusing on the role of TLRs and ROS, and therefore suggest a new therapeutic target for post-trauma ALI.

## 2. The Role of TLRs in ALI

**Figure 1:**
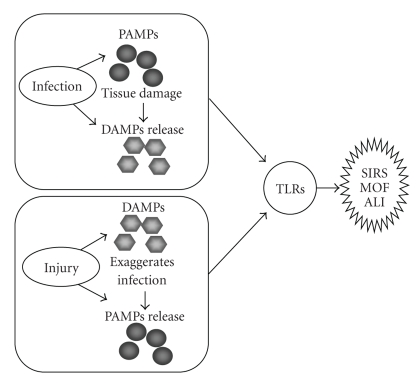
Role of TLRs in mediating inflammation and organ injury. Infection not only causes PAMPs release, but also causes tissue and cell damage along with subsequent DAMPs release. Similarly, injury caused by trauma or various other factors not only leads to DAMPs release but also renders the patient more susceptible to infection and therefore PAMPs release. In turn, PAMPs and DAMPs act through TLRs to activate the innate immune system, yet they can also contribute to persistent and deleterious systemic inflammation and organ injury, including ALI.

Toll-like receptors (TLRs) are the first family of pattern recognition receptors (PRRs) discovered in mammals. It is now well accepted that in addition to recognizing pathogen-associated molecular pattern molecules (PAMPs), TLRs can also respond to endogenous molecules released in response to stress, trauma, and cell damage. These molecules have been termed damage-associated molecular patterns (DAMPs) [12]. Interaction between PAMPs and DAMPs enhances the inflammatory response through TLR signaling, as illustrated in Figure 1. 

Activation of TLRs initiates two major pathways: the MyD88-dependent pathway, which is used by all TLRs except TLR3, resulting in the activation of NF-*κ*B and activator protein-1 (AP-1); and the TRIF-dependent pathway, which is initiated by TLR3 and TLR4, resulting in the activation of type I interferons (IFNs) [13–15]. TLRs are expressed on a range of immune cells including PMNs, macrophages, dendritic cells (DCs), B cells, and certain types of T cells, as well as on certain nonimmune cells, such as endothelial cells, smooth muscle cells, and epithelial cells that lie at potential sites of entry, including the skin, respiratory tract, intestinal tract, and genitourinary tracts [16, 17]. 

TLR4 and TLR2 sit at the interface of microbial and sterile inflammation by selectively responding to both bacterial products and endogenous ligands [18], including hyaluronic acid [19], heparan sulfate [20], fibrinogen [21], heat shock proteins [22], and high-mobility group box 1 (HMGB1) [23, 24]. Both inflammation and injury responses in organs subjected to ischemia/reperfusion depend, at least partially, on TLR4 and TLR2 [23–26]. Previous studies from both our group and others have demonstrated that a low level expression of TLR2 in cells can be upregulated by TLR4 signaling [27, 28], suggesting a mechanism of inducible cellular sensitivity to both exogenous and endogenous stimuli. In this paper, we will focus on the role of TLR4 and TLR2 cross talk in the mechanisms of post-trauma ALI.

### 2.1. Role of TLR4

Expression of functional TLR4 has been found in many cell types in the lung [29]. LPS-induced lethal shock and ALI are TLR4 dependent [30–32]. Findings from our laboratory have demonstrated that LPS downregulates TLR4 expression in AM*ϕ*, whereas antecedent HS prevents the decrease in TLR4 gene transcription in response to LPS; also, LPS-induced TLR4 mRNA destabilization is reduced in the AM*ϕ* exposed to HS as compared to that in sham animals [33]. These findings suggest that sustained TLR4 expression following HS may contribute to an enhanced cell response to LPS [33].

Recent studies have shown that HMGB1 is a potent activator of TLR4 [34]. The activation of TLR4 signaling by HMGB1 has been verified in cell lines [35, 36]. HMGB1 was initially identified as a nuclear protein that stabilized nucleosome formation and acted as a transcription factor regulating the expression of several genes [37]. HMGB1 is now known to be as an early inflammatory mediator in ischemia [23, 24], trauma, HS, and noninfectious hepatitis [38, 39]. Marked increase in HMGB1 levels in serum, lungs, and liver was detected within 2 h after HS in mice [40]. Regarding how HS induces HMGB1 secretion, study has shown that epinephrine directly acts through M*ϕ* *β*-adrenergic receptor to stimulate HMGB1 secretion from the M*ϕ* in an autocrine manner [41].

### 2.2. Role of TLR2

TLR2 is predominantly expressed in the cells involved in first-line host defense, including monocytes, macrophages, dendritic cells, and PMNs [27, 42]. In ECs and epithelial cells the TLR2 expression is low [27], but can be upregulated [43]. TLR2 senses a broad range of components from bacteria, mycoplasma, fungi, and viruses. These components include lipoproteins from a number of pathogens, PGN and LTA from Gram-positive bacteria, LAM from mycobacterium, glycosylphophatidylinositol anchors from *Trypanosoma Cruzi*, a phenol-soluble modulin from *Staphylococcus epidermis*, zymosan from fungi, and glycolipids from *Treponema maltophilum* [44–47]. TLR2 recognizes its ligands by forming a heterodimer with either TLR1 or TLR6. The resulting TLR1/TLR2 and TLR6/TLR2 complexes recognize distinct ligands, triacyl and diacyl lipoproteins, respectively. 

**Figure 2:**
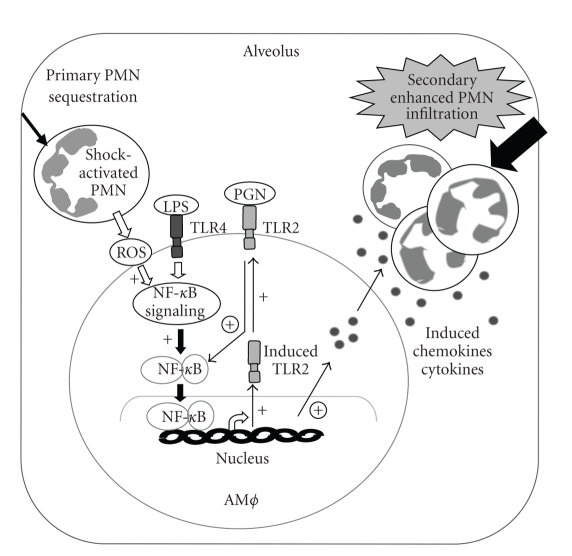
Model of shock-activated PMN in mediating the TLR4-TLR2 cross talk in AM*ϕ* and AM*ϕ* priming. Hemorrhagic shock-activated PMNs primarily migrate into alveoli in response to a trivial inflammatory stimulus, such as LPS, and interact with AM*ϕ*. The interaction between PMN and AM*ϕ* enhances LPS-induced TLR2 expression (+) in the AM*ϕ*, possibly mediated by PMNs-derived oxidants and augmented NF-*κ*B activation. The increased TLR2 expression results in the amplified response of AM*ϕ* to the TLR2 agonist (PGN), thereby augmenting cytokines and chemokines expression (circled +) and promoting enhanced PMN transalveolar migration. Thus, the shock-activated PMN-mediated TLR4-TLR2 cross talk activates a positive feedback signal leading to AM*ϕ* priming and exaggerated lung inflammation in response to invading pathogens.

We have reported that in HS, HMGB1 through TLR4 signaling upregulates TLR2 in ECs, and this upregulation associates with an amplified EC function including augmented activation of NADPH oxidase and expression of ICAM-1 in response to TLR2 activation by HMGB1 [48]. Previous reports, using both *in vivo* HS mouse model and *in vitro* PMN-AM*ϕ* coculture approaches, have also demonstrated that TLR4 upregulates TLR2 expression in AM*ϕ*, and this upregulation is significantly augmented by HS-activated PMNs [49]. The amplified TLR4-induced TLR2 expression in AM*ϕ* serves as an important mechanism underlying HS-primed lung inflammation in response to a second challenge from bacterial products. The study shows that upregulated TLR2 markedly increases expression of macrophage inflammatory protein-2 (MIP-2), cytokine migration inhibitory factor (MIF), and TNF-*α* in the AM*ϕ* and induces augmented PMN migration in response to TLR2 ligand PGN (Figure 2) [49]. 

The inducible expression of TLR2 suggests an important physiological significance of TLR-TLR cooperativity, namely that as ligand activation of TLR4 signaling wanes, the signaling functions can be transferred to TLR2, and thus the TLR mediated cellular response can be maintained over a prolonged period of time [28, 50].

## 3. The Role of ROS in ALI

ROS is a collective term that includes a large variety of free oxygen radicals, for example, superoxide anion (O_2_^•−^) and hydroxyl radicals (^•^OH), as well as derivatives of oxygen that do not contain unpaired electrons, such as hydrogen peroxide (H_2_O_2_), hypochlorous acid (HOCl), peroxynitrite (ONOO), and ozone (O_3_) [51]. During normal cellular metabolism, ROS are steadily produced. However, recent reports have demonstrated their involvement in signaling which affects cellular functions including gene expression, proliferation, cell death, migration, and inflammation [52]. ROS are generated from various catalytic pathways mediated by enzymes which are differentially localized inside the cell, including NO synthases, enzymes of the respiratory chain, cytochrome P450 monoxygenases, xanthine oxidase, and NADPH oxidase. 

Studies have suggested that ischemia/reperfusion primes circulating PMNs for increased ROS production, thereby augmenting PMN-mediated lung injury once the PMNs are sequestered in the lung [53, 54]. ROS appear to participate in the regulation of TLR4 gene expression. The use of the antioxidant *N*-acetylcysteine (NAC) supplementation during resuscitation markedly reduced levels of TLR4 mRNA and partially reverses the prolongation of TLR4 mRNA half-life observed following HS [33]. 

### 3.1. NADPH Oxidase is an Important Source of ROS in HS

Emerging evidence has shown that ROS derived from NADPH oxidase play an important role in mediating organ injury after HS [55–57]. The NADPH oxidase complex presents in a variety of phagocytic and nonphagocytic cells. The phagocytic NADPH oxidase serves a critical function in host defense against invading microorganisms. However, the nonphagocytic NADPH oxidase has been thought to mainly induce oxidant signaling, although oxidant generation is markedly less in nonphagocytic cells [58]. NADPH oxidase is a group of multimeric enzymes whose activity results in the production of O_2_^−^. NADPH oxidase consists of 5 subunits: p40*^phox^*, p47*^phox^, *p67*^phox^*, p22*^phox^*, and gp91^*pho*x^. In the basal state, p40*^phox^*, p47*^phox^, *and p67*^phox^* exist in the cytosol as a complex, while p22*^phox^* and gp91*^phox^* are located in the membranes of secretory vesicles and specific granules of PMN, where they aggregate to form a heterodimeric flavohemoprotein known as cytochrome b_558_. Upon stimulation, the cytosolic component p47*^phox^* is phosphorylated and the entire cytosolic complex migrates to the membrane where it associates with cytochrome b_558_ to assemble the active oxidase [58]. Although all of the ROS-producing enzymes contribute to the oxidative burden, NADPH oxidase seems to be a key source of ROS, and evidence has shown that an initial generation of ROS by NADPH oxidase triggers the release of ROS by other enzymes [59].

**Figure 3:**
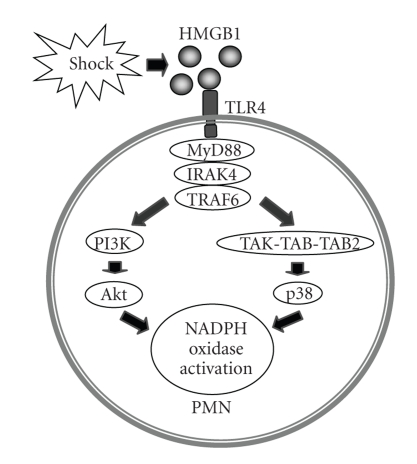
Model of HS-induced PMN NADPH oxidase activation. HMGB1 acts through TLR4 and MyD88-dependent signaling to mediate HS-induced NADPH oxidase activation. Akt and P38 MAP kinase are both involved in this event.

It has been demonstrated that HS through HMGB1 activates the TLR4-MyD88-IRAK4 signaling pathway and further activates p38 MAPK and Akt pathways to initiate phosphorylation of p47*^phox^* and subsequent activation of NADPH oxidase (Figure 3) [2]. The ROS derived from PMN NADPH oxidase not only play an important role in enhancing TLR2 upregulation in AM*ϕ* and ECs, as described below, but also contribute to endothelial NADPH oxidase activation in HS [48]. The study shows that oxidant signaling by the PMN NADPH oxidase enhances the activation of EC NADPH oxidase in response to HS through a signaling pathway involving HMGB1, TLR4, and Rac1, but independent of p38 MAPK [48].

### 3.2. Role of ROS in TLR2 Upregulation in AM*ϕ*

We have reported an important role of PMN NADPH oxidase in mediating amplified LPS-induced TLR2 upregulation in AM*ϕ* [40]. Using both *in vivo* hemorrhage mouse model and *in vitro* PMN-AM*ϕ* coculture approaches, the studies demonstrated that the TLR4-dependent TLR2 upregulation in AM*ϕ* is significantly augmented by antecedent shock; and this effect of shock is particularly mediated by shock-induced ROS released from PMN. The endogenous NADPH oxidase in AM*ϕ* may also be involved in the signal transduction, however, the exogenous ROS from PMN NADPH oxidase are essential for inducing amplified TLR2 expression in AM*ϕ* in response to TLR4 signaling, because when the AM*ϕ* from NADPH oxidase-deficient gp91^*phox*−/−^ mice were cocultured with PMNs isolated from WT mice subjected to shock, the expression of TLR2 in the gp91^*phox*−/−^ AM*ϕ* was elevated to the same level as that in WT AM*ϕ* [40]. Figure 3 illustrates the physiological significance of the ROS-augmented TLR2 upregulation by TLR4 in AM*ϕ*.

### 3.3. Role of ROS in TLR2 Upregulation in Lung EC

**Figure 4:**
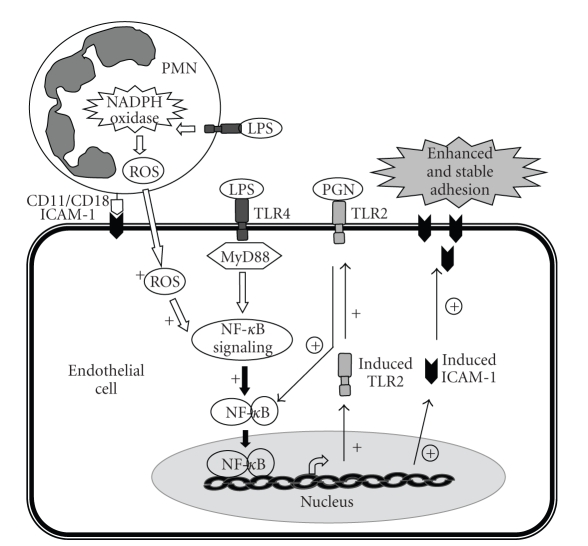
Role of PMN NADPH oxidase-derived oxidant signaling in mediating the TLR4-TLR2 cross talk in ECs. LPS stimulation induces NADPH oxidase activation and production of reactive oxygen species (ROS) in PMN as well as the initiation of MyD88-dependent NF-*κ*B signaling in ECs and the consequent expression of TLR2 and ICAM-1. Adhesion of PMN to ECs is mediated by the binding of constitutive ICAM-1 to CD18 integrin and provides the appropriate coupling required for PMN to transmit oxidant signals to ECs. The oxidants augment NF-*κ*B signaling and TLR2 expression (+), which result in the augmented response of the cell to PGN, thereby amplifying ICAM-1 expression (circled +) and promoting stable adhesion of PMN to ECs and increased PMN migration. Thus, the PMN NADPH oxidase-mediated TLR4-TLR2 cross talk activates a positive feedback signal leading to sustained and amplified endothelial activation in response to invading pathogens.

ROS also contribute to LPS/TLR4 signaling induced TLR2 expression in lung EC [28]. LPS through TLR4-MyD88-dependent signaling activated NF-*κ*B and induced TLR2 expression in ECs, and this process was enhanced by oxidant signaling generated by PMN NADPH oxidase. The functional relevance of NADPH oxidase in mediating TLR4-induced TLR2 expression in ECs was evident by markedly elevated and stable ICAM-1 expression as well as augmented PMN migration in response to sequential challenge with LPS and PGN (Figure 4) [28].

Interaction of PMNs with ECs is important for the process of PMN sequestration into the lung [30, 60]. EC activation and expression of adhesion molecules are critical to initiate a firm ICAM-1-dependent PMN adhesion to EC and, thus, mediate the early-onset migration of PMNs across the endothelial barrier. *In vivo* PMN depletion and repletion experiments demonstrated that PMN NADPH oxidase is an important determinant of TNF*α*-induced NF-*κ*B activation and ICAM-1 expression in lung EC [61]. *In vitro* PMN-EC coculture study also showed that WT PMNs induced a rapid and augmented increase in ICAM-1 expression in lung ECs from WT and NADPH oxidase-deficient p47^*phox*−/−^ mice in response to TNF*α* stimulation; while, antioxidant GSH prevented the effect of WT PMNs in amplifying ICAM-1 expression in the ECs, indicating that the interaction between PMNs and ECs is mediated through PMN-derived ROS [62].

## 4. Targeting Both TLRs and ROS as a Novel Therapeutic Strategy for ALI

As described above, ROS derived from PMN NADPH oxidase through interaction between PMNs and AM*ϕ* or PMNs and lung ECs mediate an augmented upregulation of TLR2 in the AM*ϕ* and ECs following HS, and in turn, sensitize the cells to TLR2 agonists, exaggerate inflammatory response, and promote the development of ALI. Based on these findings, targeting both TLRs and ROS simultaneously may present a novel therapeutic strategy for ALI. 

Drugs targeting TLRs mainly include either agonists of TLRs to enhance immune responses against infectious agent, or antagonists designed to reduce inflammation due to infection or autoimmune responses [63]. Since TLR4 deficiency displayed a beneficial effect in attenuating inflammation following trauma, HS, and ischemia/reperfusion [23, 48, 64, 65], TLR antagonists should be considered during the treatment of post-trauma ALI. The approaches to reduce TLR activity have focused on two aspects: (a) monoclonal antibodies, soluble receptors, and other accessory proteins and (b) signal transduction blockers. For instance, a natural soluble form of TLR2 has been found in mouse plasma and breast milk that acts to block TLR2 ligand stimulation [66]. In addition, a member of the TLR/IL-1 receptor family, TIR8 or SIGIRR, has been described to inhibit NF-*κ*B signaling and suggested an endogenous inhibitor of the TLR system [67]. Many of the key molecules in the signaling pathways for each TLR have been identified and are considered druggable targets [43, 68, 69]. The structural basis of the TIR domain of TLRs and adapters, such as MyD88, Mal, TRAM, or TRIF, has been modeled, and small peptidic sequences based on the TIR domain BB loop or peptidomimetics of this region have been made that can block the interactions [69–71]. Although the drugs targeting TLRs have not yet been applied to the treatment of ALI, the critical role of TLRs in the development of ALI targeting of PRRs has opened up a productive area for the therapy of ALI.

ROS-induced injury was considered to occur by at least two major mechanisms: (a) a direct toxic effect of reactive oxygen species on cellular components including lipid peroxidation, oxidation of critical protein thiols on enzymes, and structural protein and nucleic acid damage [72]; (b) an indirect effect mediated via activation of cell signaling pathways culminating in the generation of a number of proinflammatory molecules [73]. The role of ROS in augmenting TLRs crosstalk is now a new addition to the mechanisms underlying ROS-induced organ injury. Moreover, ROS overwhelm the endogenous antioxidant mechanisms, thereby rendering tissues more susceptible to oxidant damage. Indeed, excessive oxidative stress has been shown to correlate with poor outcomes in patients with acute respiratory distress syndrome [74, 75]. Therefore, one potential strategy for obviating the effects of oxidative stress is to employ antioxidant strategies aimed at neutralizing oxidants or enhancing endogenous antioxidant mechanisms. NAC combines both of these properties. NAC can exert antioxidant activity through a direct scavenger effect by virtue of its reduced sulfhydryl group and also by entering the cell and releasing cysteine, which contributes to the synthesis of intracellular reduced glutathione, an important regulator of the intracellular redox balance [76]. This drug is also attractive for use in humans, since there is long-standing experience with its use in the management of acetaminophen toxicity [77]. In fact, some human studies including those employing NAC as the antioxidant have shown antioxidant therapy to be beneficial [78–81]. Effective NADPH oxidase inhibitors also have been investigated in the attempt to salvage organs from oxidative injury. Two chemically distinct inhibitors of NADPH oxidase, namely diphenylene iodonium (DPI) and 4-hydroxy-3-methoxy-acetophenone (apocynin), have been found to reduce the organ injury associated with HS [55]. Apocynin in combination with TNF-*α* converting enzyme inhibitor (TACEI) can completely prevent lung from ischemic injury [61]. This system may represent an effective therapeutic approach for the delivery of antioxidants and other anti-inflammatory treatments into the lung after HS.

## 5. Conclusion

The current pharmacotherapy has not been highly successful in increasing survival during ALI. The role of TLR signaling in the development of ALI has now been well recognized. Recent studies have further demonstrated that ROS are important determinants for augmented TLR4-induced TLR2 upregulation in HS. Therefore, antioxidant strategies together with modification of TLR pathways are likely to be a logical therapeutic target for ALI.
